# The Essential Network (TEN): Protocol for an Implementation Study of a Digital-First Mental Health Solution for Australian Health Care Workers During COVID-19

**DOI:** 10.2196/34601

**Published:** 2022-03-09

**Authors:** Matthew James Coleshill, Peter Baldwin, Melissa Black, Jill Newby, Tanya Shrestha, Sam Haffar, Llewellyn Mills, Andrew Stensel, Nicole Cockayne, Jon Tennant, Samuel Harvey, Helen Christensen

**Affiliations:** 1 Black Dog Institute Sydney Australia; 2 University of New South Wales Medicine University of New South Wales Sydney Australia; 3 School of Psychology University of New South Wales Sydney Australia; 4 Discipline of Addiction Medicine Faculty Medicine and Health University of Sydney Sydney Australia

**Keywords:** blended care, mental health, burnout, health care workers, COVID-19, health care service, health service

## Abstract

**Background:**

The COVID-19 pandemic has placed health care workers (HCWs) under severe stress, compounded by barriers to seeking mental health support among HCWs. The Essential Network (TEN) is a blend of digital and person-to-person (blended care) mental health support services for HCWs, funded by the Australian Federal Department of Health as part of their national COVID-19 response strategy. TEN is designed as both a preventative measure and treatment for common mental health problems faced by HCWs. New blended services need to demonstrate improvements in mental health symptoms and test acceptability in their target audience, as well as review implementation strategies to improve engagement.

**Objective:**

The primary objective of this implementation study is to design and test an implementation strategy to improve uptake of TEN. The secondary objectives are examining the acceptability of TEN among HCWs, changes in mental health outcomes associated with the use of TEN, and reductions in mental health stigma among HCWs following the use of TEN.

**Methods:**

The implementation study contains 3 components: (1) a consultation study with up to 39 stakeholders or researchers with implementation experience to design an implementation strategy, (1) a longitudinal observational study of at least 105 HCWs to examine the acceptability of TEN and the effectiveness of TEN at 1 and 6 months in improving mental health (as assessed by the Distress Questionnaire [DQ-5], Patient Health Questionnaire [PHQ-9], Generalized Anxiety Disorder [GAD-7], Oldenburg Burnout Inventory [OBI-16], and Work and Social Adjustment Scale [WSAS]) and reducing mental health stigma (the Endorsed and Anticipated Stigma Inventory [EASI]), and (3) an implementation study where TEN service uptake analytics will be examined for 3 months before and after the introduction of the implementation strategy.

**Results:**

The implementation strategy, designed with input from the consultation and observational studies, is expected to lead to an increased number of unique visits to the TEN website in the 3 months following the introduction of the implementation strategy. The observational study is expected to observe high service acceptability. Moderate improvements to general mental health (DQ-5, WSAS) and a reduction in workplace- and treatment-related mental health stigma (EASI) between the baseline and 1-month time points are expected.

**Conclusions:**

TEN is a first-of-a-kind blended mental health service available to Australian HCWs. The results of this project have the potential to inform the implementation and development of blended care mental health services, as well as how such services can be effectively implemented during a crisis.

**International Registered Report Identifier (IRRID):**

DERR1-10.2196/34601

## Introduction

### Background

The stress of the COVID-19 pandemic is placing health care workers (HCWs) at increased risk of poor mental health [1], with posttraumatic stress disorder (PTSD) being a major concern [2-5]. Mental health treatments can lower the risk of HCWs developing mental ill-health [1], yet few appropriate services are available at the necessary scale for all specializations of HCWs. The Australian health care workforce is currently estimated at 800,000 [6]. This figure will likely continue to grow alongside population growth, indicating that large-scale solutions are required. Early in the COVID-19 pandemic, researchers called for evidence-based, self-guided mental health services for HCWs, citing reluctance to seek help, coupled with the challenges of delivering quality support to thousands of time-poor consumers [7]. Blended care services that integrate digital (websites and apps) and person-to-person (including telehealth) services [8] can rapidly scale, while offering a personalized choice of evidence-based care options [9]. In this manner, blended care is a promising means of providing large-scale mental health services to HCWs during the ongoing COVID-19 pandemic.

Over and above scalability, mental health services must be sensitive to the specialized needs of HCWs. As HCWs battle the pandemic, they face numerous sources of stress, such as fear of infection [10], unsupportive workplaces [11], and even watching colleagues die [12]. HCWs also vary in how they manage these stressors across time [13]. Therefore, HCWs need adaptable mental health services that can help with a range of concerns, from situational distress to moral injury [14] to PTSD [2,3]. Yet, even services tailored to HCWs have seen little uptake despite high reported demand [15]. One likely explanation is the lack of psychological safety many HCWs experience when accessing mental health care. Concerns about stigma [1,16], confidentiality, and discrimination from colleagues or employers [17,18] keep many HCWs from using the available services. Blended care can address some of these concerns through digital services (eg, anonymous chat-based therapies, accessing tailored resources without registration), while still providing the flexibility for HCWs who prefer person-to-person care to access these services.

To fully leverage the potential of blended care to support HCWs during the COVID-19 pandemic and beyond, services must be implemented in a way that ensures HCWs feel psychologically safe to use them. Thus far, little is known about implementing these services is a way that builds trust and increases uptake of available services. The few studies within the COVID era describe institution-specific implementation practices without data on the effectiveness of implementation strategies [19]. Studies implementing HCW-specific mental health services from previous pandemics are largely of poor quality and evaluate the effects of an intervention rather than implementation strategies to facilitate uptake and engagement [20]. These challenges exemplify implementation science in digital health, which is yet to produce a unified implementation framework to guide not only the reporting of strategies but also the evaluation of certain approaches [21,22]. Understanding how to effectively implement HCW-specific mental health services could boost the uptake of available care and inform the development of new services as the pandemic continues to unfold.

### The Essential Network

**Figure 1:**
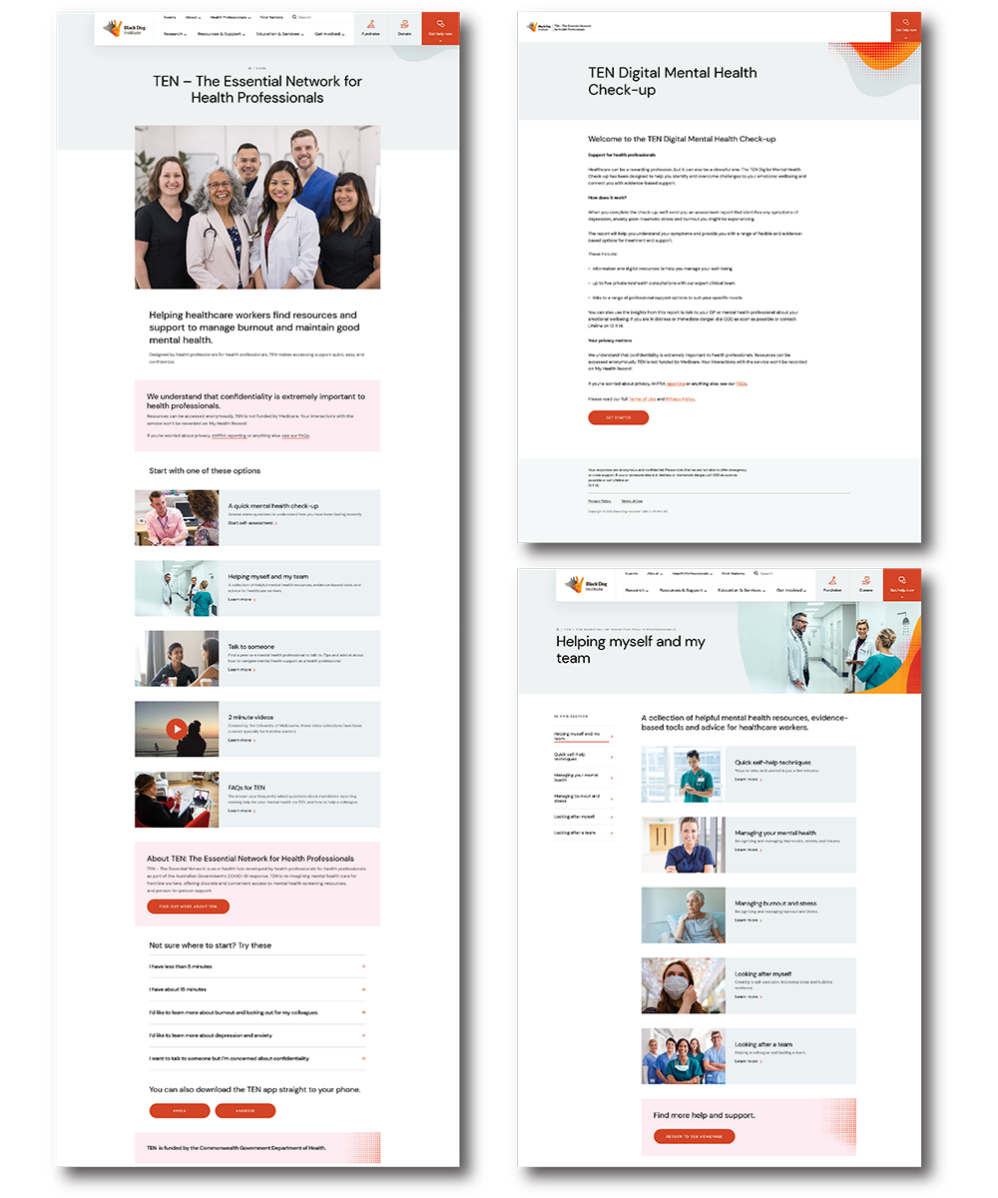
Example screenshots from the TEN website: home page (left), an online mental health assessment tool (top right), and evidence-based tools for practical self-help (bottom right). TEN: The Essential Network.

The Essential Network (TEN) was funded by the Australian Government Department of Health as part of its national COVID-19 response strategy. TEN follows a sequential blended care approach [9] using an integrated service model that spans 4 care phases: (1) well-being promotion, (2) early detection and preventative interventions, (3) low-to-moderate-intensity services for HCWs experiencing preclinical distress, and (4) person-to-person clinical services for HCWs’ mental health difficulties or clinical distress. In this sequential blended care approach, users engage with digital resources and treatment options, as required, and progress to person-to-person clinical services, where necessary. This selection of treatment options is made possible by a “digital ecosystem” that brought together a network of existing and new services from partner organizations with interest in the mental health of HCWs. Services are made available through a single web-based digital hub (Figure 1) accessible across desktop, laptop, and mobile web browsers. Digital services comprise an online mental health assessment, mental health resources and advice for assisting individuals and their colleagues, and links to other relevant resources and organizations (eg, psychiatrists who treat HCWs, peer support organizations). Person-to-person services comprise up to 5 individual clinical consultations with either a clinical psychologist or a psychiatrist delivered either via telehealth or in person at our clinic in Sydney, New South Wales (NSW), Australia. Users can select and explore the digital or person-to-person options that they believe best match their needs.

### Implementation Framework

This study will examine the implementation of TEN, a blended mental health service for Australian HCWs established during COVID-19, funded by the Australian Federal Government. The Consolidated Framework for Implementation Research (CFIR) framework will be used to guide this examination as the constructs within the CFIR span both implementation and effectiveness within a health service context [23]. However, some adaptation of the CFIR will be required. The CFIR assumes that an intervention will be implemented in a single organization or industry, unlike TEN, which spans many organizational structures and professions. Similarly, the frameworks consolidated within the CFIR are largely aimed at implementing singular, intervention-based face-to-face services, rather than multichannel digital services like TEN. Previous attempts to consolidate implementation strategies for digital mental health have focused on delivering specific digital programs as an alternative to traditional mental health services, rather than linking users to a range of care options in the way TEN does [21]. Work is underway to consolidate optimal digital health implementation strategies [22]; however, no such framework exists yet. For these reasons, in our study, the CFIR subconstruct definitions described by Damschroder et al [23] will be adapted to the digital and multiorganizational nature of TEN, guided by the digital-specific implementation strategies suggested by Graham et al [21] (see Table 1).

### Study Objectives

The primary objective of this study is to work with HCW researchers, industry partners, and active TEN users to design and test an implementation strategy for TEN. The secondary objectives are to examine the acceptability of TEN, to measure any changes in psychosocial well-being associated with the use of TEN, and to determine whether TEN reduces mental health stigma among HCWs.

**Table 1:** CFIR^a^ constructs and example interview questions.

CFIR subconstruct		Example interview question
**Intervention characteristics**		
	Intervention source	N/A^b^. TEN^c^ was externally developed.
	Evidence strength and quality	Interviews: Questions regarding perceptions of the quality of the TEN website/offering and beliefs that the care options offered will benefit the mental health of HCWs^d^.
	Relative advantage	Interviews: Questions regarding awareness of TEN equivalents and perceived advantages of TEN.
	Adaptability	Interviews: Questions regarding the capacity for TEN to be adapted to the needs of professional^e^ groups.
	Trialability	Interviews: Questions regarding the feasibility of implementation and the evaluation of TEN within a single organization or professional groups^e^.
	Complexity	Interviews: Questions regarding difficulty of implementing TEN.
	Design quality and packaging	Interviews: Questions regarding perception of the quality of TEN presentation to organizations and users^e^.
	Cost	Interviews: Questions regarding resourcing (financial, personnel, etc) and opportunity costs of using TEN.
**Outer setting**		
	Patient needs and resources	Interviews: Questions regarding knowledge of member/employee needs^e^ and how these align with the organizational strategy.
	Cosmopolitanism	Interviews: Questions regarding organizational networks.
	Peer pressure	Interviews: Questions regarding knowledge of related organizational efforts and how these might affect the organizational endorsement of TEN.
	External policies and incentives	Interviews: Questions regarding external policy considerations and how these might affect the organizational endorsement of TEN.
**Inner setting**		
	Structural characteristics	Interviews: Questions regarding the social architecture, age, maturity, and size of an organization and profession^e^.
	Networks and communications	Interviews: Questions regarding how organizations communicate with members or employees (generally and in relation to mental health).
	Culture	Interviews: Questions regarding norms, values, and basic assumptions of an organization and profession^e^.
	Implementation climate^f^	Interviews: Questions regarding current endorsement of TEN and whether TEN competes with other initiates for resources or strategic importance.
	Readiness for implementation^f^	Interviews: Questions regarding readiness of an organization to assist with the implementation strategy^e^.
**Characteristics of individuals**		
	Knowledge and beliefs about the intervention	Interviews: Questions regarding the value and knowledge of both TEN and digital/blended mental health^e^.
	Self-efficacy	Interviews: Questions regarding the capabilities of both stakeholders and members/employees^e^ to execute implementation strategies.
	Individual stage of change	Interviews: Questions regarding the stage of change most members/employees^e^ are likely in with respect to using TEN or blended mental health services^e^.
	Individual identification with organization	Interviews: Questions regarding how stakeholders perceive the relationship between their organization and members/employees^e^.
	Other personal attributes	Interviews: Questions regarding what personal traits, such as tolerance of ambiguity, intellectual ability, motivation, values, competence, capacity, and learning styles, of their members/employees^e^ are likely to affect the implementation of TEN.

^a^CFIR: Consolidated Framework for Implementation Research.

^b^N/A: not applicable.

^c^TEN: The Essential Network.

^d^HCW: health care worker.

^e^Language adapted to the multiorganizational context of TEN.

^f^Adaptation of original CFIR definitions to TEN.

## Methods

### Study Design

**Figure 2:**
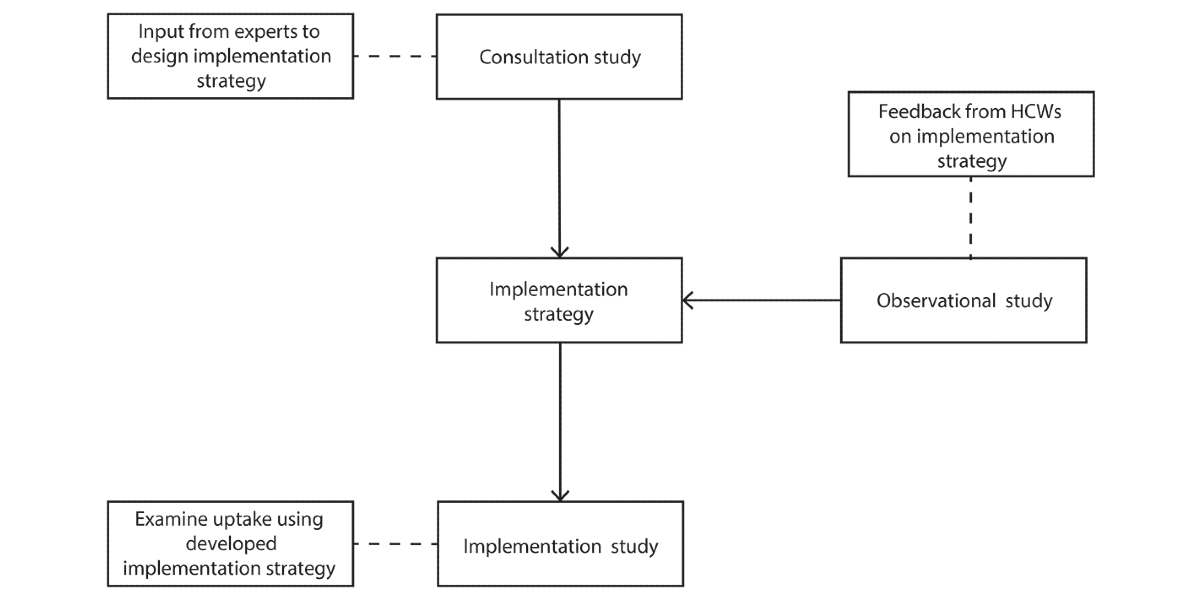
Flowchart depicting the design and components of the implementation trial. HCW: health care worker.

This study will follow an implementation-effectiveness hybrid design [24]. The protocol has 3 components (Figure 2). The *consultation study* component (Study 1) is a series of semistructured qualitative interviews conducted with stakeholders to explore the needs of Australian HCWs and develop an implementation strategy for TEN. The *observational study* component (Study 2) is a pre-/postobservational study with Australian HCWs evaluating the acceptability TEN, providing feedback on the implementation strategy, and examining any changes in mental health outcomes that are associated with use of the service. The *implementation study* (Study 3) component will be a pre-/postaudit of TEN users both before and after the implementation strategy is implemented [25].

### Study Setting

There is no specific study site. Semistructured interviews carried out as part of the consultations will be conducted online via videocalls (eg, Zoom, Microsoft Teams). As a digital mental health hub, participants in the observational study will be able to participate in the study and access the TEN service from any location in Australia. If applicable, face-to-face consultations through the TEN Clinic may be conducted at the Black Dog Institute, Randwick, NSW 2031, or via telehealth.

### Study 1: Consultation Study

#### Ethics

The consultation component received approval from the UNSW Human Research Ethics Panel (HC no. 3500) on June 7, 2021.

#### Participants

Participants in the consultation study will be representatives from Australian professional organizations (*n*=15-18) relevant to mental health or HCWs as well as researchers or staff from the Black Dog Institute (*n*=10-21) working on projects relevant to HCWs’ mental health.

#### Recruitment

Participants for the consultation component will be approached directly to discuss participation or emailed an invitation to the study and provided with an online participant information statement and consent form.

#### Data Collection

Qualitative data will be captured in semistructured interviews with HCW researchers and industry partners who represent the interests and views of their respective disciplines. Interview questions will be structured to elicit information directly relevant to the key constructs constituting the CFIR and derived from the subconstruct descriptions provided by Damschroder et al [23]. Where appropriate, questions regarding some CFIR subconstructs will be adapted to a multiorganizational or digital context.

### Study 2: Observational Study

#### Ethics

The observational component received approval from the UNSW Human Research Ethics Committee (HC no. 210394) on July 22, 2021.

#### Participants

Participants in the observational study will be self-identified Australian HCWs who currently reside in Australia and have sufficient English proficiency to participate. Participant status as HCWs will be verified using the Australian Health Practitioner Regulation Agency’s register of practitioners. Participants who cannot be verified using the register of practitioners will be contacted on a case-by-case basis to confirm eligibility.

#### Recruitment

Participants for the observational study will be recruited through advertisements on social media, emails sent to health professionals on an internal Black Dog Institute mailing list, emails from TEN partner organizations, and information about the study on the Black Dog Institute and TEN websites. Prospective participants who engage with advertisements or study details on the TEN website will be redirected to the online participant information statement and consent form. Participants who elect not to participate in the study will be provided with a link to TEN and informed they may use the service freely and anonymously.

#### Data Collection

**Figure 3:**
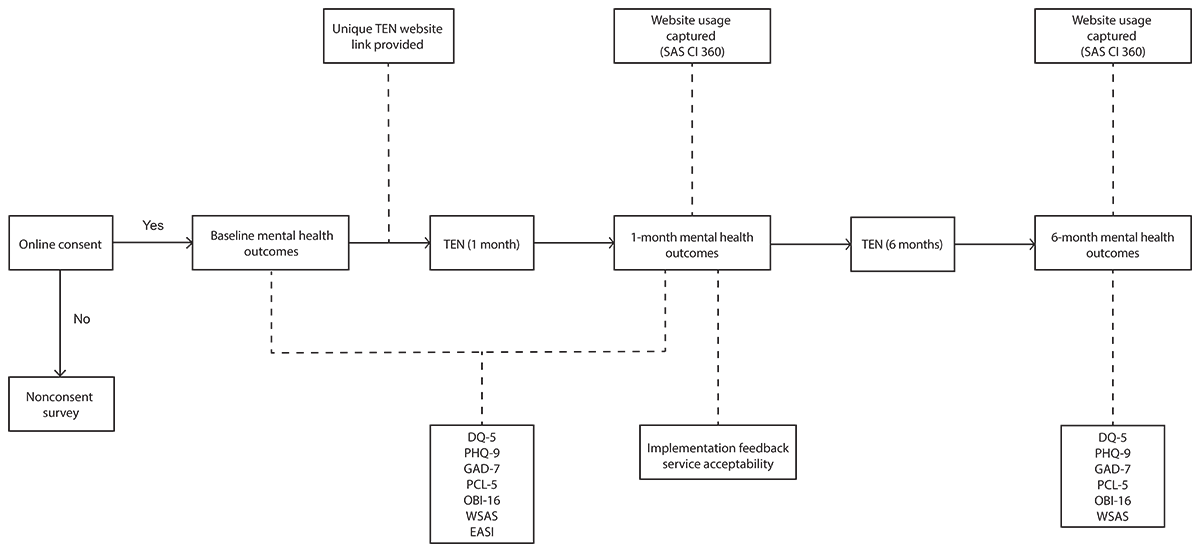
Flowchart depicting a progression through the observational study. DQ-5: Distress Questionnaire; EASI: Endorsed and Anticipated Stigma Inventory; GAD-7: Generalized Anxiety Disorder; OBI-16: Oldenburg Burnout Inventory; PCL-5: Posttraumatic Stress Disorder Checklist; PHQ-9: Patient Health Questionnaire; SAS CI 360: SAS Customer Intelligence 360; TEN: The Essential Network; WSAS: Work and Social Adjustment Scale.

Quantitative data will be collected through an observational study with Australian HCWs (Figure 3). Participants will provide online consent using the e-Consent feature of REDCap (Vanderbilt University). People who decline to participate will be provided with a link to TEN and asked to complete a brief survey asking why they opted not to participate.

After consenting, participants will provide information on demographics and mental health (Table 2), service acceptability (Table 3), and complete baseline mental health and stigma outcomes (Table 4). Mental health outcomes are identical to the TEN online assessment. To avoid the participants needing to repeat the online assessment on the TEN website after completing the baseline mental health outcomes, the survey baseline will provide the same assessment feedback. Participants will then be provided with a link to the TEN website and asked to engage with the service naturalistically for 6 months. The link provided to each participant is unique and associated with their consent record number. User engagement (eg, pages viewed) with the TEN website is recorded automatically through the SAS Customer Intelligence 360 (SAS CI 360) platform. SAS CI 360 is a commercial website analytics package that records website use. Using unique TEN website links sent to participants, their TEN website use will be extracted and linked to their other study data. Participants will be recommended to bookmark this link, and their unique link will be provided to them by email at baseline and after the 1-month follow-up.

**Table 2:** Questions examining demographic and baseline mental health.

Baseline question	Time point	Classification	Type of question
Gender	Baseline	Demographics	Single selection
Age	Baseline	Demographics	Single selection
Where are you located?	Baseline	Demographics	Single selection
What best describes your profession?	Baseline	Demographics	Single selection
Have you previously used the TEN^a^ service?	Baseline	Mental health	Yes/no
COVID-19 has affected your mental health.	Baseline	Mental health	Likert scale
Have you ever seen a doctor, counsellor, or other health professional about your mental health?	Baseline	Mental health	Yes/no
If you were seeking help for a mental health problem, what type of service would you most likely choose?	Baseline	Mental health	Multiple selection
Which of the following were the most important reasons why you chose to participate in this study?	Baseline	Mental health	Multiple selection
Which parts of TEN are you most interested in?	Baseline	Mental health	Multiple selection
What is your current employment status as an HCW^b^?	6 months	Employment	Single selection

^a^TEN: The Essential Network.

^b^HCW: health care worker.

**Table 3:** Questions examining TEN^a^ service acceptability.

Service acceptability question	Time point	Type of question
If you were seeking help for a mental health problem, what type of service would you most likely choose?	Baseline	Multiple selection
Which of the following were the most important reasons why you chose to participate in this study?	Baseline	Multiple selection
Which parts of TEN are you most interested in?	Baseline	Multiple selection
If you needed to, would you use the TEN Clinic if there was a service charge?	1 month	Yes/no
If you needed to, would you use the TEN Clinic if it was provided under Medicare?	1 month	Yes/no
TEN met my mental health and well-being needs.	1 month	Likert scale
TEN improved my awareness of my mental health.	1 month	Likert scale
TEN improved my psychological coping skills.	1 month	Likert scale
A mental health service I found through TEN improved my psychological coping skills.	1 month	Likert scale
I would recommend TEN to a colleague.	1 month	Likert scale
Which parts of TEN were most useful to you?	1 month	Multiple selection
Why did you find these parts of TEN useful?	1 month	Free text
Did TEN make it easier for you to find the mental health support you wanted?	1 month	Yes/no
Did TEN help you learn about new sources of support?	1 month	Yes/no
Did you have any other feedback about TEN?	1 month	Free text

^a^TEN: The Essential Network.

**Table 4:** Mental health and psychosocial instruments.

Questionnaire	Outcome
DQ-5^a^	Psychological distress
PHQ-9^b^	Depression
GAD-7^c^	Anxiety
PCL-5^d^	PTSD^e^
OBI-16^f^	Burnout
WSAS^g^	Social- and work-related impairment
EASI^h^	Mental health stigma

^a^DQ-5: Distress Questionnaire.

^b^PHQ-9: Patient Health Questionnaire.

^c^GAD-7: Generalized Anxiety Disorder.

^d^PCL-5: Posttraumatic Stress Disorder Checklist.

^e^PTSD: posttraumatic stress disorder.

^f^OBI-16: Oldenburg Burnout Inventory.

^g^WSAS: Work and Social Adjustment Scale.

^h^EASI: Endorsed and Anticipated Stigma Inventory.

After using TEN for 1 month, participants will be provided with a survey containing service acceptability (Table 3), mental health and stigma outcomes (Table 4), feedback on the implementation strategy (Table 5), and self-reported service engagement (Table 5). After completing the survey, participants will be informed that their TEN website usage will be monitored for a further 5 months to examine persistence with the service. After 6 months, participants will be provided with a follow-up survey about employment (Table 2) and mental health outcomes (Table 4).

**Table 5:** Questions examining the implementation strategy and service engagement.

Question		Type of question
**Implementation feedback**		
	Would you agree that this would be an effective strategy for engaging most HCWs^a^ with TEN^b^?	Likert scale
	Would you agree that this would be an effective strategy for engaging your specific health care profession with TEN?	Likert scale
	Do you have any thoughts about this strategy or suggestions for other strategies to engage more HCWs with TEN?	Free text
**Service engagement**		
	How much did you use the TEN service over the month?	Likert scale
	Roughly how many times would you say you accessed TEN over the month?	Numerical

^a^HCW: health care worker.

^b^TEN: The Essential Network.

#### TEN Service Acceptability

Service acceptability will be examined through survey questions provided to participants at both the 0- and 1-month time points. The questions will examine whether TEN met the participants’ mental health needs and which TEN services they found most useful (Table 3). People who decline to participate in the study will be prompted with an optional survey with a single question asking them why they opted not to participate in the study.

#### Mental Health and Psychosocial Outcomes

Mental health and psychosocial outcomes will be examined through surveys provided to participants at the 0-, 1-, and 6-month time points. The questionnaires used will examine a range of outcomes, including the Distress Questionnaire (DQ-5) [25], the Patient Health Questionnaire (PHQ-9) [26], Generalized Anxiety Disorder (GAD-7) [27], the Posttraumatic Stress Disorder Checklist (PCL-5) [28], the Oldenburg Burnout Inventory (OBI-16) [29], the Work and Social Adjustment Scale (WSAS) [30], and the Endorsed and Anticipated Stigma Inventory (EASI) [31] (Table 4). To address workplace stigma unique to HCWs, a question addressing mandatory reporting concerns was added to the workplace stigma subscale of the EASI. Except for the EASI, these questionnaires are identical to those used in the TEN online assessments. Completed online assessments are automatically collected and stored on University of New South Wales (UNSW) servers. Any completed online assessments will be linked back to the participants using their internet protocol (IP) address collected during online consent.

#### TEN Implementation Feedback

Feedback on the strategy will be examined through survey questions provided to observational study participants at the 1-month time point. Participants will first be presented with a brief overview of the implementation strategy. The questions after this overview will examine whether the implementation strategy is an effective strategy for engaging HCWs, as well as open-ended feedback on the implementation strategy (Table 5).

#### TEN Service Engagement

Engagement with TEN will be examined through survey questions provided to participants at the 1-month time point, as well as through website and service analytics. The questions will examine self-reported usage of TEN (Table 5). Website user analytics are automatically collected through the SAS CI 360 platform whenever a user interacts with the TEN website. These user analytics include the IP address of the user as well as which pages or resources were accessed and when. User analytics will be linked to the participant using the IP address provided during consent. TEN Clinic clinicians will also provide data from person-to-person clinical services, including whether a participant accessed the TEN Clinic and the number of sessions.

#### Sample Size

Sample size calculations were based on the primary analysis: a random intercepts mixed effects model estimating change in WSAS scores from baseline to 1 month in a single group of participants. Estimated scores at baseline were derived from 249 TEN mental health assessments performed between June 28 and August 28, 2021. These scores represent HCWs self-selecting to engage with the TEN service and, as such, are analogous to the observational study participants at baseline. The mean of scores in this group was 14.9 (SD 8.9). The cut-off score for a clinical case on the WSAS is 11; thus, to reduce from the mean untreated score of 14.9 to a subclinical score requires a 3.87-point reduction. With variance-covariance of random effects derived from the SD in WSAS scores from current TEN users of 8.9, and accounting for 20% attrition, a sample size of at least 105 is required to detect a true reduction of this size with 80% power and a significance level of α=.05.

### Study 3: Implementation Study

#### Ethics

Ethics approval for the implementation study will be sought from the UNSW Human Research Ethics Committee following design of the implementation strategy.

#### Participants

Participants in the implementation study will be self-identified Australian HCWs.

#### Data Collection

Quantitative data will be collected through an audit of usage of the TEN website and TEN Clinic for a period of 3 months both before and after the implementation of the implementation strategy. Although the exact implementation strategy will be determined by the results of the consultation study, example strategies from the literature include identifying “champions” within an organization to drive uptake or develop incentives, such as continuing professional development activities [32]. TEN website data include completed online assessments automatically recorded on UNSW servers and website analytics (eg, number of unique users, pages viewed) automatically collected through the SAS CI 360 platform. TEN Clinic data include service analytics data, which are routinely captured by clinicians in the delivery of TEN Clinic services. Surface-level nonidentifiable TEN Clinic data will be examined as part of the implementation study (eg, number of referrals, number of consultations).

## Results

### Study 1: Consultation Study

#### Data Analysis

A framework analysis using the CFIR will be conducted to thematically analyze and interpret the semistructured interviews from the consultations. Each transcript will be analyzed separately by 2 investigators who will meet periodically throughout data collection to discuss emerging themes and resolve discrepancies. The sample size is expected to be sufficient for thematic saturation [33].

### Study 2: Observational Study

#### Data Analysis

Descriptive statistics will be used to report the overall acceptability of TEN. The results from the observational study will be analyzed using linear mixed models to examine changes in reported psychosocial benefits over time (ie, baseline, 1 month). Patterns of service engagement and baseline outcome severity will be classified using cluster analyses or latent class analyses. These patterns will be used to examine how service engagement influences outcomes, as well as stratify participants baseline outcomes for subset analysis. Kaplan-Meier curves with TEN engagement data will be used to examine persistence with TEN over the course of the 6-month follow-up (ie, when participants ceased accessing the service). Missing data will be addressed using maximum likelihood estimation. This will answer the research questions by examining the acceptability of TEN among HCWs, patterns of service usage over time, any benefits observed after engaging with the TEN service, and persistence with the service over time.

### Study 3: Implementation Study

#### Data Analysis

Descriptive statistics will be used to report usage of the TEN website and TEN Clinic over the 3-month period both pre- and postimplementation strategy. The *t* test will be used to compare overall usage of the TEN service for these 2 periods. Linear mixed models will examine latent growth in service usage following the implementation strategy.

## Discussion

### Summary

Blended digital and person-to-person care has significant potential to support mental health of HCWs through service flexibility and widespread availability. To date, however, the potential of blended care has been hampered by a lack of knowledge around the optimal implementation of such services. The CFIR framework provides a structured approach to implementation that will guide the development and application of implementation strategies for TEN. Using a implementation-effectiveness hybrid study, this implementation study will span 3 separate components. Through a series of consultations, an implementation strategy tailored to HCWs will be developed. Feedback on this strategy will be provided by HCWs participating in an observational study of TEN, from which data on the effectiveness of TEN will be acquired. Finally, the implementation strategy will be implemented and compared against the previous engagement strategies. The knowledge created by this project will inform the development and delivery of blended mental health services for HCWs, including how such services can be rapidly launched and implemented during times of crisis.

### Potential Limitations

Although the implementation strategy has not yet been designed, the wide availability of TEN makes it difficult to examine site- or specialization-specific implementation strategies or service upgrades. Although the goal of TEN is to provide a blended care service for all Australian HCWs, this lack of specificity may hamper implementation within specific HCW specializations. The future of blended health is platforms that can be easily customized to different contexts, allowing for tailored implementation strategies and service upgrades. Similarly, the broad aims of the implementation of TEN may not be easily generalizable to other, more specific, contexts.

This implementation study and observational study will also be carried out during the ongoing COVID-19 pandemic. TEN itself was designed to support the mental health of HCWs during the pandemic. As such, changes in the COVID-19 context in Australia, such as outbreaks, restrictions, and the vaccination rollout, are likely to affect both uptake of the service and mental health outcomes. Such wider events will be considered when interpreting the results of both the implementation strategy and the observational study.

The observational study is also limited by a lack of an appropriate control arm and use of randomization. Common mental health outcomes, such as distress, anxiety, and depression, have the potential to improve over time without intervention. Although subgroup analyses examining usage of the service may shed some light on improvement without the use of the TEN service, the fact remains that a randomized controlled trial design with a suitable control would provide more robust data on efficacy.

Due to common concerns around mandatory reporting among HCWs [34], the TEN website was designed to be accessed anonymously (ie, without registration). Although this is a strength of the service itself, it poses problems for measuring participant engagement with the TEN website during a prospective study. The method used in the observational study—providing users with a unique website link—is error prone. Although participants are asked to bookmark the link and will be provided with the link via email, it remains that participants may simply access the TEN website without using their unique link. During data analysis, participant website analytics will be compared against self-reported usage to understand the extent of such errors.

### Conclusion

By using a blended care approach, TEN can overcome common barriers to HCWs engaging with mental health services—primarily by allowing HCWs to anonymously access digital resources. Nonetheless, it remains that many HCWs will still choose not to engage with person-to-person services due to concerns around mandatory reporting. Further, although the lack of registration to access TEN ameliorates concerns around digital privacy, it requires computer skills that some HCWs may be lacking. Although these are issues beyond the scope of this evaluation, they need to be considered and addressed when designing and implementing mental health services for HCWs.

## Abbreviations

CFIR: Consolidated Framework for Implementation Research
DQ-5: Distress Questionnaire
EASI: Endorsed and Anticipated Stigma Inventory
GAD-7: Generalized Anxiety Disorder
HCW: health care worker
IP: internet protocol
OBI-16: Oldenburg Burnout Inventory
PCL-5: Posttraumatic Stress Disorder Checklist
PHQ-9: Patient Health Questionnaire
PTSD: posttraumatic stress disorder
SAS CI 360: SAS Customer Intelligence 360
TEN: The Essential Network
UNSW: University of New South Wales
WSAS: Work and Social Adjustment Scale
